# Psychotic Illness in First-Time Mothers with No Previous Psychiatric Hospitalizations: A Population-Based Study

**DOI:** 10.1371/journal.pmed.1000013

**Published:** 2009-02-10

**Authors:** Unnur Valdimarsdóttir, Christina M Hultman, Bernard Harlow, Sven Cnattingius, Pär Sparén

**Affiliations:** 1 Department of Medical Epidemiology and Biostatistics, Karolinska Institutet, Stockholm, Sweden; 2 Centre of Public Health Sciences, Faculty of Medicine, University of Iceland, Reykjavík, Iceland; 3 Department of Neuroscience, Psychiatry, Ulleråker, Uppsala University, Uppsala, Sweden; 4 Department of Epidemiology and Community Health, University of Minnesota School of Public Health, Minneapolis, Minnesota, United States of America; University of Western Sydney, Australia

## Abstract

**Background:**

Psychotic illness following childbirth is a relatively rare but severe condition with unexplained etiology. The aim of this study was to investigate the impact of maternal background characteristics and obstetric factors on the risk of postpartum psychosis, specifically among mothers with no previous psychiatric hospitalizations.

**Methods and Findings:**

We investigated incidence rates and potential maternal and obstetric risk factors of psychoses after childbirth in a national cohort of women who were first-time mothers from 1983 through 2000 (*n* = 745,596). Proportional hazard regression models were used to estimate relative risks of psychoses during and after the first 90 d postpartum, among mothers without any previous psychiatric hospitalization and among all mothers. Within 90 d after delivery, 892 women (1.2 per 1,000 births; 4.84 per 1,000 person-years) were hospitalized due to psychoses and 436 of these (0.6 per 1,000 births; 2.38 per 1,000 person-years) had not previously been hospitalized for any psychiatric disorder. During follow-up after the 90 d postpartum period, the corresponding incidence rates per 1,000 person-years were reduced to 0.65 for all women and 0.49 for women not previously hospitalized. During (but not after) the first 90 d postpartum the risk of psychoses among women without any previous psychiatric hospitalization was independently affected by: maternal age (35 y or older versus 19 y or younger; hazard ratio 2.4, 95% confidence interval [CI] 1.2 to 4.7); high birth weight (≥ 4,500 g; hazard ratio 0.3, 95% CI 0.1 to 1.0); and diabetes (hazard ratio 0).

**Conclusions:**

The incidence of psychotic illness peaks immediately following a first childbirth, and almost 50% of the cases are women without any previous psychiatric hospitalization. High maternal age increases the risk while diabetes and high birth weight are associated with reduced risk of first-onset psychoses, distinctly during the postpartum period.

## Abstract 2

Unnur Valdimarsdóttir and colleagues studied the risk factors for psychiatric illness following childbirth and found that, for women who had never previously been hospitalized for a psychiatric illness, the risk of mental illness was greatly increased following childbirth.

## Editors' Summary

**Background.:**

The first cries of a new life echo around the delivery suite: this is a time of great joy for most women. Yet, in the following days and weeks (the postpartum period), up to 80% of new mothers experience some sort of mental disturbance. Usually, this is the “baby blues,” a normal reaction to childbirth that is characterized by short-lived mood swings or postnatal depression. However, about one in 1,000 women develop postpartum psychosis, a serious mental disorder that needs immediate medical attention. Postpartum psychosis usually develops suddenly in the first 2–3 weeks after delivery and, like other forms of psychosis, is characterized by a loss of contact with reality. Women with postpartum psychosis may have false ideas about current events and about themselves (delusions) and see and hear things that are not there (hallucinations). They sometimes stop eating or sleeping and may become anxious and agitated. In the worst cases, they can have suicidal thoughts or even threaten their baby's life. Treatment for postpartum psychosis includes antipsychotic drugs, counseling, and hospital admission if the woman is a danger to herself or others.

**Why Was This Study Done?:**

Women with a personal or family history of psychosis have an increased risk of developing postpartum psychosis, but what causes this disorder is unknown. The rapid changes in hormone levels that occur after delivery are likely to be involved—but might social circumstances, stress, other illnesses, or the birth itself also affect whether a woman develops postpartum psychosis? If additional risk factors for postpartum psychosis could be identified, it might be possible to prevent some cases of this serious mental disorder. In this study, the researchers investigate the incidence rate (the rate at which new cases occur in a population) and risk factors for psychotic illnesses diagnosed among first-time mothers registered in the Swedish Medical Birth Registry between 1983 and 2000.

**What Did the Researchers Do and Find?:**

The researchers identified three-quarters of a million first-time mothers and, from the Swedish Hospital Discharge Registry, found that 892 of these women (1.2 per 1,000 births) had been admitted to hospital because of psychosis within 90 days of giving birth. Put another way, the incidence rate of psychosis over the first 90 days postpartum in this population was 4.84 per 1,000 person-years. Almost half of the women who developed postpartum psychosis had not been previously admitted to hospital for any psychiatric disorder. Among this subset of women, the incidence rate of postpartum psychosis was highest during the first month after delivery but dropped to less than a tenth of this initial rate after 90 days postpartum. Furthermore, the risk of developing psychosis during the first 90 days postpartum (but not after) increased with age—women older than 35 years were more than twice as likely to develop psychosis than those aged 19 years or less—but was reduced in women who had large babies or who had diabetes. Many other factors (including smoking and not living with the infant's father) did not affect the risk of psychosis during the first 90 days postpartum in these women.

**What Do These Findings Mean?:**

These findings indicate that the occurrence of psychotic illness severe enough to require hospitalization peaks shortly after giving birth for the first time, even in women with no previous psychiatric illness. Indeed, women with no history of mental disorders account for almost half the women admitted to hospital for postpartum psychosis, at least in Sweden. The timing of the peak of postpartum psychosis supports the idea that either giving birth or the hormonal changes that occur shortly after may trigger the development of psychosis, and the findings that maternal diabetes and high infant birth weight reduce the risk of postpartum psychosis whereas increasing maternal age increases the risk provide new clues about the causes of postpartum psychosis. Most importantly, however, these findings highlight the importance of carefully monitoring women for psychosis during the first month after delivery.

**Additional Information.:**

Please access these Web sites via the online version of this summary at http://dx.doi.org/10.1371/journal.pmed.1000013.

- This paper is further discussed in a *PLoS Medicine* Perspective by Phillipa Hay
- The MedlinePlus Encyclopedia contains a page on MedlinePlus encyclopedia  psychosis (in English and Spanish); MedlinePlus also provides links to information on psychotic disorders
- The UK National Health Service Direct Health encyclopedia has information on psychosis and on postnatal depression
- Mental Health America has a fact sheet on postpartum disorders

## Introduction

A psychotic illness starting shortly after childbirth is a relatively rare condition [1,2]. However, the negative implications of such an illness can be enormous: repeated episodes of psychoses and hospitalization of the mother [3], increased risk of self-harm or suicide [4], as well as the rare but tragic occurrences of harm to the newborn infant and infanticide [5]. An obvious obstacle for effective prevention is that the etiology of psychotic illness in the postpartum period is poorly understood.

Whether the postpartum period really poses any additional risk of psychoses has been a matter of speculation: compared to population- or prepregnancy rates, relative risks from 1.09 [2] to 12.7 [1] for psychoses during the 3 mo postpartum have been reported. Apart from the mother's own or family history of psychoses [6,7], few background factors have consistently been associated with increased risks of psychoses in the postpartum period [8]. In a previous study we found that almost 14% of women with previous psychiatric hospitalizations suffered from postpartum psychotic or bipolar episodes compared to 0.05% of women without previous psychiatric hospitalizations [7]. Still, some women have their first and sometimes their only psychotic episode during the postpartum period [9,10]. It is not known if this is solely due to a biological vulnerability for psychoses, or if psychosocial or obstetric factors influence the risk. To date, few studies have addressed the influence of the mother's background and obstetric characteristics on the risk of postpartum psychosis while controlling for previous psychiatric vulnerability, e.g., psychiatric hospitalizations. Moreover, it remains to be investigated whether episodes of psychosis during the 90 d postpartum period have risk factors distinct from psychoses occurring at later times during motherhood.

Studying risk factors for psychoses during the postpartum period is a methodological challenge, because of low incidence rates and the confounding effects of previous psychiatric morbidity. Sweden has excellent conditions for research in this area, with population-based registers covering essentially all births and inpatient records. Using these data sources, we investigated the incidence rate and risk factors of psychotic illnesses diagnosed during and after the first 90 d postpartum among all Swedish first-time mothers, and specifically among mothers without any previous psychiatric hospitalization. Controlling for previous psychiatric hospitalizations, we hypothesized that established risk factors for nonpuerperal psychosis as well as obstetric complications would increase the risk of maternal psychoses during the first 90 d postpartum.

## Methods

### Population

All primiparous women registered in the Swedish Medical Birth Registry from 1 January 1983 to 31 December 2000 (*n* = 745,596) were considered. Diseases in Swedish registers are coded according to the International Classification of Diseases (ICD); the 8th version (ICD-8) was used through 1986, the 9th revision (ICD-9) between 1987 and 1996, and the 10th revision (ICD-10) has been used since 1997. Women without any registered psychiatric diagnosis (ICD-8 codes 290–390; ICD-9 codes 290–316; and ICD-10 codes F00–F99) in the Hospital Discharge Registry before the date of delivery were analyzed separately.

### Psychoses Criteria during the Postpartum Period

We included information on psychoses registered in the Hospital Discharge Registry as a primary or secondary diagnoses (ICD-8 codes 294.4, 295–299; ICD-9 codes 295–298; and ICD-10 codes F20–31 or F53.1) during the first 90 d postpartum; or alternatively, later than 90 d until next pregnancy, emigration from Sweden, death, or end of follow-up (31 December 2001). A psychosis case could have had more than one hospitalization for psychoses after childbirth, but we counted only the first hospitalization. The discharge diagnoses are made by the attending psychiatrist based on observations during hospitalization and evaluation of the patient as well as medical records at discharge. The registered ICD discharge diagnoses have been reported to have high agreement with diagnoses based on DSM (Diagnostic and Statistical Manual of Mental Disorders) criteria [10] as well as with diagnoses based on semistructured interviews and medical records [11].

Women hospitalized for psychoses were categorized into the following diagnoses: (1) postpartum psychosis (ICD-8 code 294.4; ICD-10 code F53.1), (2) acute/reactive psychosis (ICD-8 codes 298.00–299.99; ICD-9 codes 298A–X; ICD-10 codes F23 and F28–29), (3) schizophrenia (ICD-8 codes 295.00–295.99; ICD-9 codes 295A–X; ICD-10 code F20), (4) affective (ICD-8 codes 296.00–296.99; ICD-9 codes 296A–X; ICD-10 codes F30–3), (5) paranoia (ICD-8 codes 297.00–297.98; ICD-9 codes 297BCDWX; ICD-10 code F22) and, (6) schizoaffective disorder (ICD-8 code 295.7; ICD-9 code 295H; ICD-10 code F25).

### Potential Risk Factors

Independent variables were mainly obtained from the Medical Birth Registry. The maternal background factors were: age at birth; years of education (from the Education Registry); family situation (living with child's father or not); country of birth (Sweden; other Nordic country; non-Nordic European country, North America, Australia and New Zealand; Asia, Africa, South America); living in one of the three biggest cities in Sweden (Stockholm, Gothenburg, and Malmö) or not; hypertensive disease (ICD-8 codes 400–404 or 637; ICD-9 code 642; ICD-10 codes O10–O14); diabetes (pregestational or gestational; ICD-8 code 250; ICD-9 codes 648A or 648W; ICD-10 code O24); maternal cigarette smoking at admission to maternity care; and year of birth.

Obstetric and perinatal exposures were: perinatal death (stillbirth or infant death within 7 d), congenital malformations (ICD-8 and ICD-9 codes 740–759, ICD-10 codes Q00–Q99), gestational age (in completed weeks), and birth weight. Large for gestational age (LGA) and small for gestational age (SGA) were defined as a birth weight of more or less than 2 standard deviations from the mean birth weight for gestational age, respectively, according to the Swedish reference curve for fetal growth [12]. We also included information on the infant's sex, single or multiple births (the index child being the first-born multiple), mode of delivery (vaginal or cesarean delivery), dystocia (ICD-8 code 657; ICD-9 code 661; ICD-10 code O62), and antepartum bleeding (ICD-8 codes 632 and 651; ICD-9 code 641; ICD-10 codes O44–O46).

The study conforms to STROBE guidelines for cohort studies (Text S1) and was approved by the Ethics Review Board at Karolinska Institutet, # 03–462.

### Analyses

We calculated the proportion of cases with no previous psychiatric hospitalizations from the total psychosis cases during the first 90 d after first births. The incidence rates of total psychosis cases and first psychosis cases were calculated within categories of 30 d of the first year postpartum and then annually until next pregnancy, death, emigration from Sweden, or towards the end of the observation period (31 December 2001); whichever occurred first. We used Cox's proportional hazard regression models to calculate adjusted hazard ratios for psychoses (95% confidence intervals [CIs]) during and after the first 90 d postpartum. The Wald test for interactions was performed to test potential interactions between previous psychiatric hospitalizations and all maternal as well as obstetric characteristics on the risk of psychosis during the first 90 d postpartum. SAS systems software version 9.1 was used for the statistical analyses.

## Results

### Incidence Rates

Of 745,596 first-time mothers, 892 women (1.2 per 1,000 births) were hospitalized for psychoses during the first 90 d postpartum. In all, 436 women (49%; 0.6 per 1,000 births) had not previously been hospitalized for any psychiatric disorder, and the majority of them were diagnosed with reactive/acute psychoses or a postpartum psychosis (Table 1).

**Table 1:**
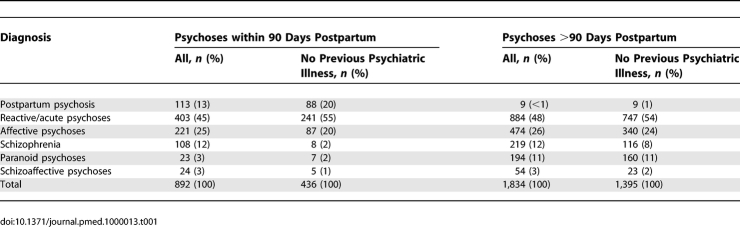
Psychosis Diagnoses of All First-Time Mothers and First-Time Mothers without Any Previous Psychiatric Hospitalizations during and after the First 90 Days Postpartum

**Figure 1:**
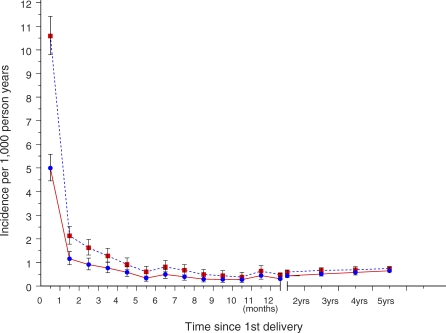
Incidence of Psychoses among Swedish First-Time Mothers Dashed line: all maternal psychoses; solid line: psychoses in mothers without any previous psychiatric diagnoses.

Incidence rates of maternal psychoses during the first 12 mo postpartum and then annual incidence rates until the end of the observation period are illustrated in Figure 1. Incidence rates peaked during the first month following childbirth; 285 out of the total 892 psychosis cases (32%) were hospitalized within 7 d after childbirth and 523 (59%) within 14 d. During the first 90 d postpartum, approximately half of the total number of psychosis cases were accounted for by women without any previous psychiatric hospitalization; the 30-d incidence rate was 4.99 (95% CI 4.46–5.58) per 1,000 person-years, the 90 d incidence rate was 2.38 (95% CI 2.17–2.62) per 1,000 person-years, and the incidence rate for the rest of the observation period was 0.49 (95% CI 0.46–0.52) per 1,000 person-years.

### Maternal Characteristics

Table 2 shows hazard ratios during and after 90 d postpartum for all maternal psychoses as well as for women without any previous psychiatric hospitalization, adjusted for maternal characteristics.

**Table 2:**
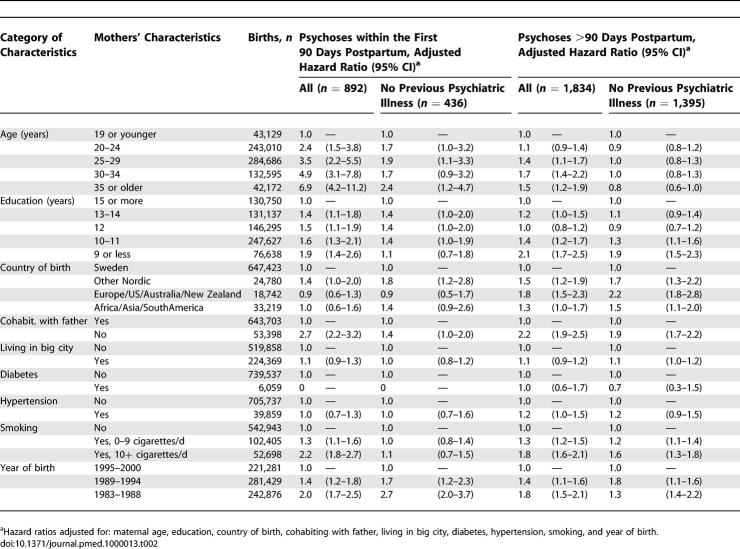
Swedish First-Time Mothers' Characteristics and Health as Potential Risk Factors for Psychoses during and after the First 90 Days Postpartum

Maternal age and diabetes specifically affect the risk of psychoses during the first 90 d postpartum. Higher maternal age increases the risk of psychoses among mothers without any previous psychiatric hospitalization during the first 90 d postpartum. Compared to mothers aged 19 y or younger, mothers aged 35 y or older had a more than two-fold increased risk of psychoses during, but not after, the first 90 d postpartum.

More than 6,000 women were diagnosed with diabetes (gestational or pregestational); none of these women developed postpartum psychoses during the first 90 d postpartum, while the corresponding risk after the 90 d was around 1.0.

In contrast to all women, women without any previous psychiatric hospitalization had none or a limited increase in risk of psychoses during the first 90 d postpartum due to low level of education, not cohabitating with the infant's father, and maternal smoking. However, these factors had similar impact on the risk of psychoses after the first 90 d postpartum among all women as well as among women without any previous psychiatric hospitalization. The increased risk of psychoses among immigrant women (the mother being born outside Sweden), particularly among mothers born in another Nordic country, was amplified after the first 90 d postpartum. Psychosis hospitalizations of all women, before and after the 90 d postpartum period, decreased markedly in Sweden during the observation period (Table 2).

When we added perinatal death to the model presented in Table 2, the hazard ratio of psychoses for women 35 y or more and without any previous psychiatric hospitalizations decreased from 2.4 to 1.9 (95% CI 0.9–4.0) during the first 90 d postpartum.

### Pregnancy and Obstetric Characteristics

Women without any previous psychiatric hospitalization had an increased risk of psychoses when giving birth to a child with very low birth weight (≤ 1,500 g). In contrast, high birth weight (≥ 4,500 g) and LGA decreased the risk by 70%–80% during, but not after, the first 90 d postpartum (Table 3). Perinatal death, congenital malformations, preterm birth (<32 wk), and cesarean delivery were not statistically significant risk factors of psychoses among women without any previous psychiatric hospitalization. However, these adverse pregnancy outcomes increased the risk of psychoses among all women during the first 90 d postpartum. After the first 90 d postpartum, perinatal death was the only obstetric factor increasing the risk of psychoses among all women, and multiple births showed a protective effect for psychoses among mothers without any previous psychiatric hospitalization as well as for all mothers.

**Table 3:**
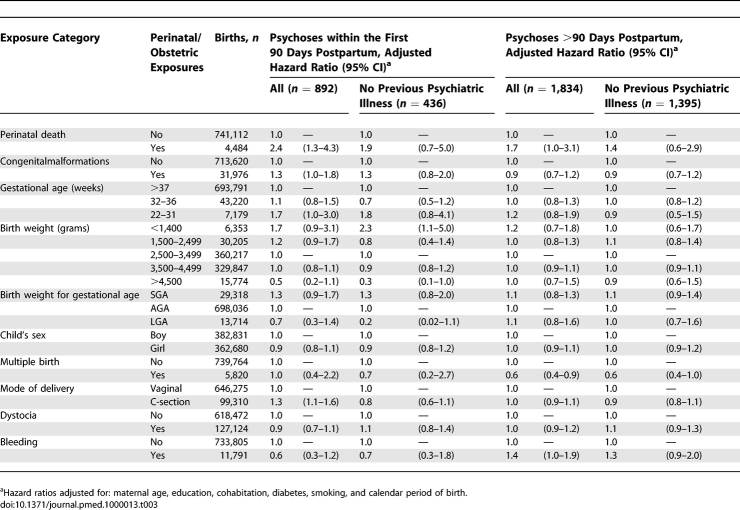
Exposures Related to the Child or Obstetric Complications and the Risk of Psychoses during First 90 Days Versus 91 Days or Later Postpartum among Swedish First-Time Mothers

The Wald test for interactions was performed to test potential interactions between previous psychiatric hospitalizations and all maternal as well as obstetric characteristics on the risk of psychosis during the first 90 d postpartum. No statistically significant interactions were observed except for cesarean section (*p* = 0.0015); maternal age (*p* = 0.0830) and gestational age (*p* = 0.0949) were the only other variables approaching (but not reaching) statistical significance. We further tested for interactions between maternal age and other maternal characteristics on the risk of postpartum psychosis; none of these proved statistically significant.

## Discussion

Our findings suggest that the immediate time period following childbirth entails (compared to subsequent periods) a substantially increased risk of psychotic illness of the first-time mother. This also holds true for mothers without any previous psychiatric hospitalization that account for almost half of the psychosis cases during the first 90 d postpartum. Among women without any previous psychiatric hospitalization, greater maternal age and lower birth weight of the infant increase the risk of psychoses distinctly during the postpartum period, while maternal diabetes and high birth weight of the infant appear to be protective. These findings have implications for the etiology of psychotic illness occurring in the immediate postpartum period.

### The Incidence of Psychoses during the Postpartum Period

Women without any previous psychiatric hospitalizations before the date of delivery had a more than ten times higher incidence rate during the first month postpartum compared to after the first 90 d postpartum. Thus, our findings provide strong support for the notion that the period following childbirth carries an increased risk of psychoses among women without any previous psychiatric hospitalization. Whether the rate of first psychosis episodes in first-time mothers reaches the levels of nulliparous women following the 90 d postpartum remains to be investigated. Population rates of first psychoses among women represent a complicated comparison to our study since these typically reflect rates of nulliparous, primiparous, and multiparous women during and after the postpartum period.

The fact that one-third of all the psychosis cases during the first 90 d are concentrated in the first 7 d (one-twelfth of the total period) indicates that the birth may have a causal role. As suggested by previous research [1,13] stressful characteristics of the birth, e.g., perinatal death, might be a triggering factor. Alternatively, women after childbirth experience an enormous decrease in levels of estrogens and other hormones produced by the placenta. The hypothesis that dramatic reductions in hormone exposure mediate the risk of psychoses after childbirth has some support from previous research. Firstly, the higher age of onset of schizophrenia in women as compared to men, and the increased female perimenopausal incidence rates of schizophrenia have been suggested to be related to loss of the protective effects of estrogens [14]. Secondly, schizophrenic women may be more likely to be admitted for their psychoses during the low-estrogen phase of their menstrual cycle [15]. Thirdly, improvements of postpartum psychotic symptoms after estradiol administration were reported in a case series of about dozen mothers [16,17]. In contrast, no protective effects were found of estradiol administration on a postpartum relapse of 29 women previously diagnosed with affective psychoses [18]. Thus, the evidence is not conclusive on whether a change in estrogen exposure or other hormones around childbirth may be a triggering factor for psychoses during the postpartum period.

### Postpartum-Specific Risk Factors

The investigation of risk factors for maternal psychoses following childbirth is complicated by the risk of confounding by previous psychiatric illness. We therefore specifically studied potential maternal and obstetric risk factors among women without any previous psychiatric hospitalization.

We found that maternal diabetes and high infant birth weight seemed protective of first-onset psychoses during, but not after, the first 90 d postpartum. We are not aware of any previous study reporting such findings. Regarding diabetes, the possibility of a chance finding cannot be excluded; however, similar findings were reported in a German study: in 313 people newly diagnosed with type 1 diabetes, none had a probable nonpuerperal psychotic disorder as compared to 1.5% in representative comparison sample of 2,046 individuals [19]. Intense surveillance of diabetic mothers during pregnancy might reduce the risk of overt psychotic illness: a recent report indicates that such a program for diabetic mothers reduces the risk of adverse pregnancy outcomes as well as risk of depression 3 mo postpartum [20]. Alternatively, diabetic mothers tend to have larger babies [21], and high birth weight was also associated with protection from psychoses during the first 90 d postpartum. Pregnancies involving large babies [22] as well as multiple births [23] have both been associated with high levels of estrogen. However, pregnancy hormones are produced by the placenta and hormone levels decrease rapidly after the expulsion of the placenta. Thus, although possible, it still remains uncertain whether the possible protective mechanisms of diabetes or high birth weight include hormonal effects.

The majority of previous studies addressing mean maternal age [1,9,24,25] or the proportion of cases within different age groups [13] have not found age differences between women with and without psychoses during the postpartum period. Similar to our findings on all first-time mothers, a recent Swedish study observed that mothers aged 40–44 y faced a more than 6-fold increased risk of psychoses during the postpartum period compared to mothers aged 20–24 y [26]. These age-related risks may be exaggerated by confounding effects of uncontrolled previous psychiatric morbidity; when we excluded women with any kind of previous psychiatric hospitalizations, we found that older women (≥ 35 y) still had a more than 2-fold increased risk of psychoses during the first 90 d postpartum.

The fact that the relationship between maternal age and psychoses is restricted to the first 90 d indicates that our findings are reasonably not explained by a “natural” age of onset of psychotic disorders. Further, previous findings suggest that the onset of nonpuerperal psychotic disorders peaks at a somewhat different age level than was observed in the present study among first-time mothers [14,27]. Moreover, the hypothesis that the association is explained by the notion that undetected “premorbid women” delay their childbearing is also undermined by the specific “postpartum nature” of the relationship. The notion that the effect of maternal age is mediated by hormonal differences remains uncertain: some have found estrogen levels during pregnancy to vary with maternal age [28] while others have not [22]. Alternatively, the risk of adverse pregnancy outcomes, e.g., perinatal death, has been reported to increase with maternal age [29,30]. Probably because of small numbers, perinatal death was not statistically significantly associated with psychoses among women without any previous psychiatric hospitalizations, and the age-related risk was reduced in an additional analysis when including perinatal death as a covariate. Thus, it is possible that such severe obstetric stress partly mediates the relationship between maternal age and the risk of psychoses.

Our findings provide no evidence that the relationship between severe obstetric hazards (perinatal death or congenital malformation) and postpartum psychosis is different for all mothers versus mothers without previous psychiatric hospitalizations. While a previous investigation reported an increased risk of postpartum psychosis after cesarean delivery [13], our interaction tests show that cesarean delivery is associated with increased risk for postpartum psychoses only among women with previous psychiatric hospitalizations, whereas there is a tendency towards protection among women without previous psychiatric hospitalization.

In contrast to the postpartum-specific risk factors, our findings indicate that not cohabitating with the child's father, maternal smoking, and lower maternal education are general risk factors for maternal psychoses; it is unlikely that these factors have any causal role for psychoses that are limited to the immediate postpartum period. Further, we observed an increased risk of psychoses among mothers born outside of Sweden. Being born in another Nordic country was a risk factor for maternal psychoses during and after the first 90 d postpartum. Additional analyses among the women born in other Nordic countries revealed that a Finnish nationality carried the highest risks (unpublished data). A previous Swedish study indicates that after controlling for sociodemographic factors, elevated relative risks of psychotic conditions remain high among first- and second-generation Finnish immigrants [31].

### Validity

Based on a nationwide cohort of 745,596 first-time mothers, to our knowledge our study is the largest to date to address risk factors of maternal psychosis during the postpartum period. Strengths of the study include the use of complete population-based registers of births [32] and hospital discharges [10] with high validity, exploration of the influence of potential risk factors of psychoses by time, and the separate analyses of mothers without any previous psychiatric hospitalizations. Information on pre- and postpartum psychiatric disorders was limited to inpatients, and no information is available about women with clinical or subclinical syndromes who are treated as outpatients. The 50% decrease in psychosis hospitalizations during the last two decades probably reflects reforms of psychiatric care in Sweden rather than an actual decrease in incidence [33]. Consequently, the incidence rates of psychoses after childbirth are probably underestimated. Concerning the postpartum-specific risk factors, we judge the risk of potential confounding due to undetected psychotic outpatients as minimal, since the hazard ratios stay intact when controlling for calendar period of birth. However, we cannot exclude the possibility that some unmeasured confounders, e.g., social characteristics or sources of support, may explain our findings.

### Conclusion and Implications

The immediate period following childbirth carries, compared to subsequent periods, high incidence rates for psychoses in first-time mothers, even among those without any previous psychiatric hospitalization. We found the risk of such first-onset psychotic illness during the 90 d postpartum period to be increased with maternal age and reduced among mothers with diabetes and those giving birth to infants with high birth weight. These findings may have implications for the etiology of psychotic disorders during the postpartum period.

## Supporting Information

Text S1 STROBE Guidelines for Reporting Cohort Studies (89 KB DOC)

## Abbreviations

CI: confidence interval
ICD: International Classification of Diseases
LGA: large for gestational age
SGA: small for gestational age

## References

[pmed-1000013-b001] Kendell RE, Chalmers JC, Platz C (1987). Epidemiology of puerperal psychoses. Br J Psychiatry.

[pmed-1000013-b002] Terp IM, Mortensen PB (1998). Post-partum psychoses. Clinical diagnoses and relative risk of admission after parturition. Br J Psychiatry.

[pmed-1000013-b003] Terp IM, Engholm G, Moller H, Mortensen PB (1999). A follow-up study of postpartum psychoses: prognosis and risk factors for readmission. Acta Psychiatr Scand.

[pmed-1000013-b004] Appleby L, Mortensen PB, Faragher EB (1998). Suicide and other causes of mortality after post-partum psychiatric admission. Br J Psychiatry.

[pmed-1000013-b005] Spinelli MG (2001). A systematic investigation of 16 cases of neonaticide. Am J Psychiatry.

[pmed-1000013-b006] Jones I, Craddock N (2001). Familiality of the puerperal trigger in bipolar disorder: results of a family study. Am J Psychiatry.

[pmed-1000013-b007] Harlow BL, Vitonis AF, Sparen P, Cnattingius S, Joffe H (2007). Incidence of hospitalizations for postpartum psychotic and biopolar episodes in women with and without prior pre-pregnancy or prenatal psychiatric hospitalizations. Arch Gen Psychiatry.

[pmed-1000013-b008] Seyfried LS, Marcus SM (2003). Postpartum mood disorders. Int Rev Psychiatry.

[pmed-1000013-b009] Videbech P, Gouliaev G (1995). First admission with puerperal psychosis: 7–14 years of follow-up. Acta Psychiatr Scand.

[pmed-1000013-b010] Robertson E, Jones I, Haque S, Holder R, Craddock N (2005). Risk of puerperal and non-puerperal recurrence of illness following bipolar affective puerperal (post-partum) psychosis. Br J Psychiatry.

[pmed-1000013-b011] Kristjansson E, Allebeck P, Widstedt B (1987). Validity of the diagnosis of schizophrenia in a psychiatric inpatient register. Nord J Psychiatry.

[pmed-1000013-b012] Ekholm B, Ekholm A, Adolfsson R, Vares M, Osby U (2005). Evaluation of diagnostic procedures in Swedish patients with schizophrenia and related psychoses. Nord J Psychiatry.

[pmed-1000013-b013] Marsal K, Persson PH, Larsen T, Lilja H, Selbing A (1996). Intrauterine growth curves based on ultrasonically estimated foetal weights. Acta Paediatry.

[pmed-1000013-b014] Kendell RE, Rennie D, Clarke JA, Dean C (1981). The social and obstetric correlates of psychiatric admission in the puerperium. Psychol Med.

[pmed-1000013-b015] Hafner H (2003). Gender differences in schizophrenia. Psychoneuroendocrinology.

[pmed-1000013-b016] Huber TJ, Borsutzky M, Schneider U, Emrich HM (2004). Psychotic disorders and gonadal function: evidence supporting the oestrogen hypothesis. Acta Psychiatr Scand.

[pmed-1000013-b017] Ahokas A, Aito M (1999). Role of estradiol in puerperal psychosis. Psychopharmacology (Berl).

[pmed-1000013-b018] Ahokas A, Aito M, Rimon R (2000). Positive treatment effect of estradiol in postpartum psychosis: a pilot study. J Clin Psychiatry.

[pmed-1000013-b019] Kumar C, McIvor RJ, Davies T, Brown N, Papadopoulos A (2003). Estrogen administration does not reduce the rate of recurrence of affective psychosis after childbirth. J Clin Psychiatry.

[pmed-1000013-b020] Petrak F, Hardt J, Wittchen HU, Kulzer B, Hirsch A (2003). Prevalence of psychiatric disorders in an onset cohort of adults with type 1 diabetes. Diabetes Metab Res Rev.

[pmed-1000013-b021] Crowther CA, Hiller JE, Moss JR, McPhee AJ, Jeffries WS (2005). Effect of treatment of gestational diabetes mellitus on pregnancy outcomes. N Engl J Med.

[pmed-1000013-b022] Ehrenberg HM, Mercer BM, Catalano PM (2004). The influence of obesity and diabetes on the prevalence of macrosomia. Am J Obstet Gynecol.

[pmed-1000013-b023] Kaijser M, Granath F, Jacobsen G, Cnattingius S, Ekbom A (2000). Maternal pregnancy estriol levels in relation to anamnestic and fetal anthropometric data. Epidemiology.

[pmed-1000013-b024] Thomas HV, Murphy MF, Key TJ, Fentiman IS, Allen DS (1998). Pregnancy and menstrual hormone levels in mothers of twins compared to mothers of singletons. Ann Hum Biol.

[pmed-1000013-b025] Kumar R, Marks M, Platz C, Yoshida K (1995). Clinical survey of a psychiatric mother and baby unit: characteristics of 100 consecutive admissions. J Affect Disord.

[pmed-1000013-b026] Sharma V, Smith A, Khan M (2004). The relationship between duration of labour, time of delivery, and puerperal psychosis. J Affect Disord.

[pmed-1000013-b027] Nager A, Johansson LM, Sundquist K (2005). Are sociodemographic factors and year of delivery associated with hospital admission for postpartum psychosis? A study of 500,000 first-time mothers. Acta Psychiatr Scand.

[pmed-1000013-b028] Welham JL, Thomis R, McGrath JJ (2003). Age-at-first-registration and heterogeneity in affective psychoses. Aust N Z J Psychiatry.

[pmed-1000013-b029] Panagiotopoulou K, Katsouyanni K, Petridou E, Garas Y, Tzonou A (1990). Maternal age, parity, and pregnancy estrogens. Cancer Causes Control.

[pmed-1000013-b030] Cnattingius S, Forman MR, Berendes HW, Isotalo L (1992). Delayed childbearing and risk of adverse perinatal outcome. A population-based study. JAMA.

[pmed-1000013-b031] Cnattingius S, Stephansson O (2002). The epidemiology of stillbirth. Semin Perinatol.

[pmed-1000013-b032] Hjern A, Wicks S, Dalman C (2004). Social adversity contributes to high morbidity in psychoses in immigrants—a national cohort study in two generations of Swedish residents. Psychol Med.

[pmed-1000013-b033] Cnattingius S, Ericson A, Gunnarskog J, Kallen B (1990). A quality study of a medical birth registry. Scand J Soc Med.

[pmed-1000013-b034] Osby U, Hammar N, Brandt L, Wicks S, Thinsz Z (2001). Time trends in first admissions for schizophrenia and paranoid psychosis in Stockholm County, Sweden. Schizophr Res.

